# Preclinical Development of GT-14, a Novel Inhibitor of Gα_i_2 Protein: Comprehensive Evaluation of Physicochemical, Metabolic Characteristics and Tissue Distribution

**DOI:** 10.1208/s12248-025-01166-y

**Published:** 2025-12-01

**Authors:** Mahua Sarkar, Ting Du, Yuan Chen, Yen V. Maroney Lawrence, Jing Ma, Shafiq A. Khan, Adegboyega K. Oyelere, Dong Liang, Song Gao, Huan Xie

**Affiliations:** 1 Department of Pharmaceutical Sciences, College of Pharmacy and Health Sciences, Texas Southern University, 3100 Cleburne Street, Houston, Texas 77004, U.S.A.; 2 Center for Cancer Research and Therapeutic Development, Clark Atlanta University, 223 James P. Brawley Dr., Atlanta, Georgia 30314, U.S.A.; 3 School of Chemistry and Biochemistry, Parker H. Petit Institute for Bioengineering and Bioscience, Georgia Institute of Technology, 901 Atlantic Drive, Atlanta, Georgia 30318, U.S.A.

**Keywords:** gα_i_2 protein, metabolism, physico-chemical properties, preclinical development, tissue distribution

## Abstract

GT-14, identified as [(E)-4-((1-(1-methyl-1H-indol-2-yl) ethylidene)amino)phenol], is a novel inhibitor targeting the Gα_i_2 protein, which is crucial in facilitating cell migration and invasion in prostate, ovarian, and breast cancer cells. therefore a valuable target for treating metastatic castration-resistant prostate cancer (mCRPC). In this study, GT-14’s physicochemical properties, permeability, metabolic behavior, and tissue distribution were assessed. The results showed that GT-14 exhibited very slight aqueous solubility at room temperature (0.11 mg/mL) but was soluble in solvents including dimethyl sulfoxide and dimethyl acetamide, and sparingly or slightly soluble in several cosolvents. GT-14 exhibited a distinct pH-dependent solubility profile, being stable across a broad pH range (1.2–7.4) but degrading in strongly basic conditions. It exhibited high permeability (1.3 × 10^−5^ cm/s) in Caco-2 cell culture models and therefore identified as a BCS II compound. Hepatic microsomal studies revealed that GT-14 underwent Phase I metabolism, with more than 90% remaining in 60 min incubation in rat liver microsomes. A stable co-solvent formulation was developed to enable intravenous administration for pharmacokinetic studies. Previous pharmacokinetic studies showed that GT-14 exhibited biphasic disposition with a terminal plasma elimination half-life of 268.07 minutes (> 4 hours). Tissue distribution analysis indicated the highest concentration of GT-14 in the prostate, followed by the kidneys, lungs, heart, and liver. Our study presents an early-stage preclinical drug development roadmap that integrates modern technologies for efficiency and success, using GT-14 as a model compound. It showed promising characteristics, reinforcing its potential as a new therapeutic agent for mCRPC.

## Introduction

Prostate cancer is a complex disease that affects millions of men globally, predominantly in high human development index regions (1). It is the most widely diagnosed solid malignancies in males in 118 countries of the world. Worldwide, prostate cancer is the 2nd most frequent cancer, closely following lung cancer, and it’s the 5th leading cause of death from cancer (after lung, liver, colorectal, and stomach cancers) (2). According to the American Cancer Society’s estimates, approximately 313,780 new cases of prostate cancer are expected, with about 35,770 deaths anticipated in 2025 in the United States (3). A substantial proportion of these patients develop metastatic castrationresistant prostate cancer (mCRPC) that progresses clinically, radiographically, or biochemically despite castration levels of serum testosterone (4). The prognosis of mCPRC varies with age, ethnicity, genetic background, patient health condition, and the histopathological, anatomical, and molecular profiles of the tumor.

Androgen deprivation therapy (ADT), based on inhibition of androgen biosynthesis and/or action, is accepted universally as the first-line treatment of symptomatic mCRPC. It consists of luteinizing hormone-releasing hormone (LHRH) agonists, LHRH antagonists, or bilateral orchiectomy. Additionally, androgen receptor signaling inhibitors (ARSI) and chemotherapy are combined with the ADT backbone for superior patient outcomes (5). There have been constant new additions to the therapeutic landscape in mCRPC, such as new cytostatic agents, second-generation antiandrogens, bone-targeted therapies, immunotherapy, poly (adenosine diphosphate-ribose) polymerase (PARP) inhibitors, Akt inhibitors, and radioisotopes (4). Although mCRPC patients currently benefit from a wealth of effective treatment options, mCRPC remains incurable, and the prognosis of these patients remains poor. Therefore, it remains an area of active research investigation.

Studies by Zhong *et al* (2012) and Caggia *et al* (2018) have demonstrated that Gα_i_2 protein, a subunit of the heterotrimeric G-protein complex, plays an essential role in the migration and invasion of prostate, ovarian and breast cancer cells. This novel effect of Gα_i_2 makes it a promising therapeutic target for mCRPC (6, 7).

**Fig. 1:**
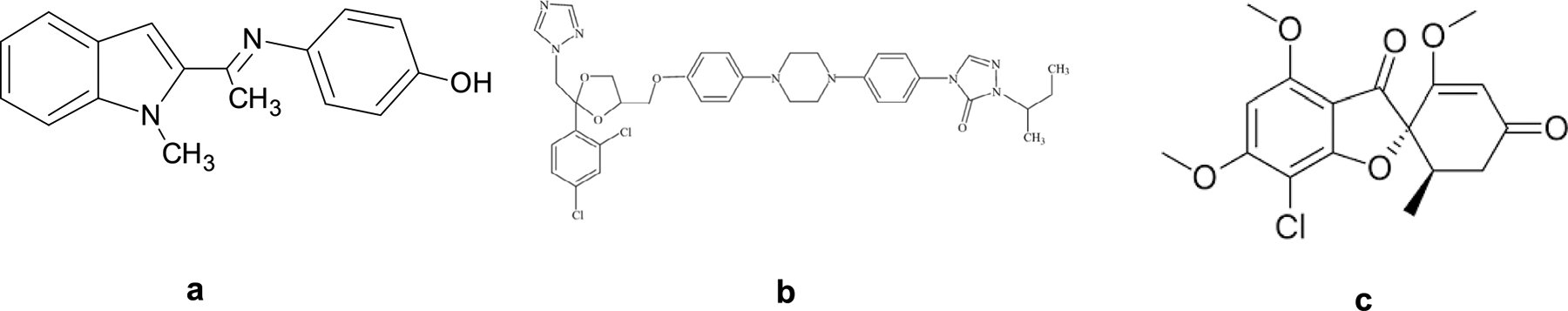
Chemical Structures of **a** GT-14 (C_17_H_17_ON_2_), and **b** Itraconazole (C_35_H_38_C_l2_N_8_O_4_) (Internal Standard for UPLC assay); **c** Griseofulvin (C_17_H_17_ClO_6_) (Internal Standard for LC-MS/MS assay)

Several small molecule inhibitors were developed and synthesized by Dr. Oyelere’s group at Georgia Tech Institute of Technology, to validate Gα_i_2 protein as a therapeutic target (8, 9). Out of the series of lead compounds tested for efficacy in Dr. Khan’s lab at Clark Atlanta University, Compound 14 (now referred to as GT-14), a first-generation inhibitor of Gα_i_2 protein, demonstrated significant reduction in the migratory capability of PC3 and DU145 prostate cancer cell lines (9). A recent study by Caggia *et al*. showed that simultaneous treatment with GT-14 blocked the stimulatory effects of docetaxel in PC3 cells, and HDACi and antiandrogen effects on cell migration in LNCaP cells. These results suggest that combination therapy with anti-androgens (or chemotherapy) and GT-14 could blunt the capability of cancer cells to migrate and may potentially block metastasis (8). GT-14 should be further explored as an anti-metastatic candidate that can be used either alone or in combination regimens with the current standards-of-care treatments for mCRPC. GT-14, identified as [(E)−4-((1-(1-methyl-1H-indol-2-yl) ethylidene)amino)phenol] (Fig. 1a), is a novel inhibitor targeting the Gα_i_2 protein. It is a phenolic imine with a molecular weight of 264.32 g/mol, predicted logP of 3.50. We have previously developed an LC-MS/MS assay with a quantification limit of 0.78 ng/mL in rat plasma suitable for biopharmaceutics and pharmacokinetic characterizations (10). However, limited preclinical drug development information is available about GT-14.

**Fig. 2:**
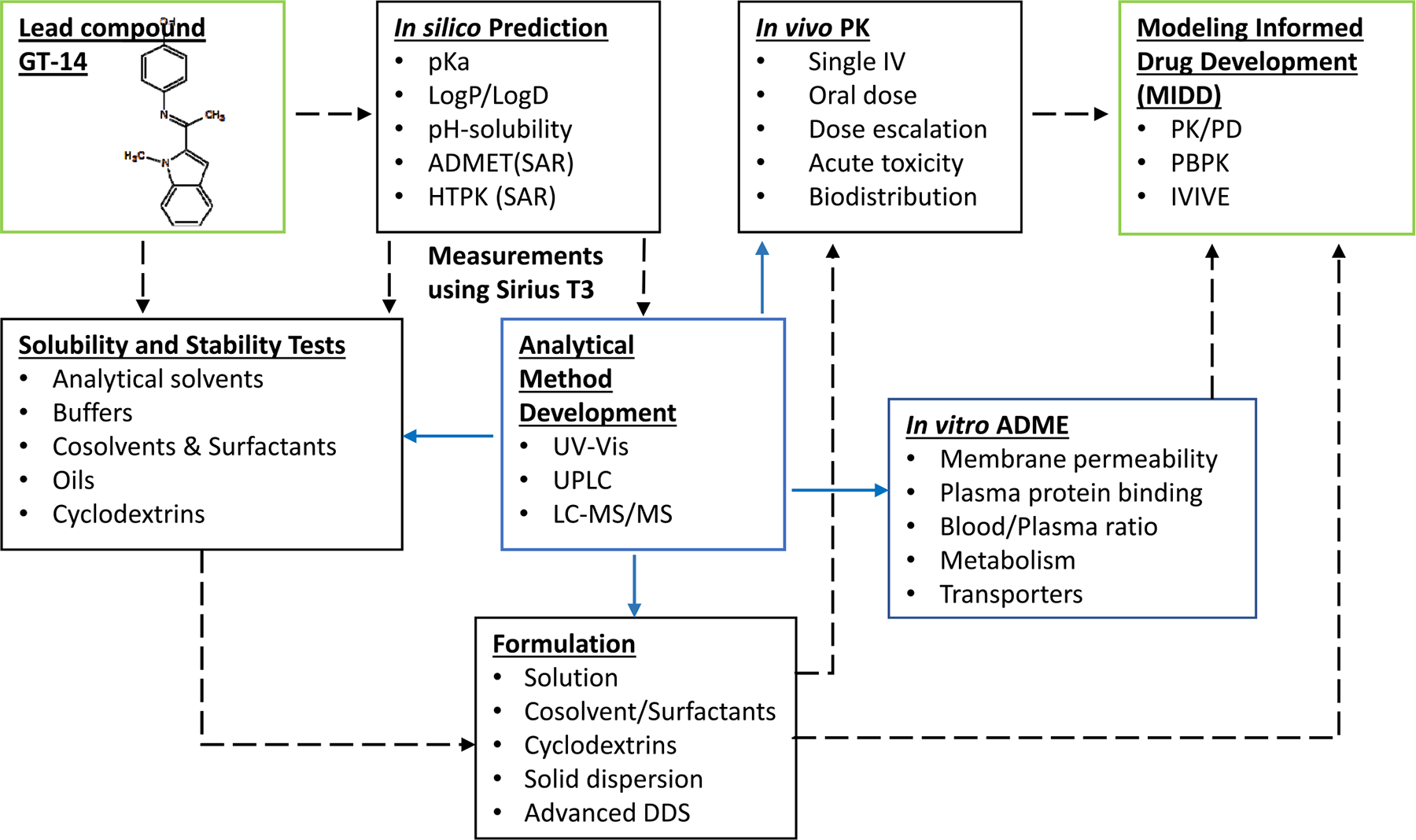
Roadmap of preclinical development for Investigational New Drug (IND) at Institute of Drug Discovery and Development (iD^3^) at Texas Southern University (TSU). The black dashed arrows indicate the sequential progression of the experimental workflow, while the solid blue arrows represent the generation and integration of quantitative data at each stage of development

At our Institute of Drug Discovery and Development (iD^3^) at Texas Southern University (TSU), we follow a roadmap to develop novel drugs and bridge the gap in drug discovery from preclinical to Investigational New Drug (IND) filing (Fig. 2). The preclinical development of a lead molecule begins with *in silico* prediction, which estimates physicochemical properties (pKa, logP, pH-solubility, and ADMET profile). These predictions help guide both formulation development and analytical method development. Next, experimental measurements (pKa, logP, solubility determination) are conducted to inform the selection of suitable formulations for *in vivo* studies. Concurrently, robust analytical methods (UPLC, LC-MS/MS) are developed and validated for accurate quantification of the molecule in biological samples. *In vitro* ADME (absorption, distribution, metabolism, and excretion) studies are performed to assess the compound’s cellular permeability, plasma protein binding, metabolic pathways and stability. Finally, *in vivo* pharmacokinetic (PK) studies in animal models evaluate ADME, providing critical data to support dose selection and further development.

In this study, using GT-14 as a model novel drug candidate, we followed the strategic roadmap, conducted most of the studies outlined in Fig. 2, and applied “go” or “nogo” decision criteria process in early-stage preclinical drug development. We performed a comprehensive evaluation of GT-14 by characterizing its physicochemical properties (solubility, stability, lipophilicity), Caco-2 cell permeability, hepatic microsomal metabolic stability and biotransformation, and *in vivo* PK and tissue distribution. The GT-14 development tracked the roadmap and promoted this novel compound closer to becoming an anti-metastatic agent for patients with advanced mCRPC.

## Materials and Methods

### Materials

GT-14, [(*E*)−4-((1-(1-methyl-1*H*-indol-2-yl) ethylidene) amino)phenol] (>95% purity), was custom synthesized by Molport (Riga, Latvia). Solvents such as dimethyl acetamide (DMA), dimethyl sulfoxide (DMSO), ethanol, propylene glycol, polyethylene glycol 300 (PEG 300), polyethylene glycol 400 (PEG 400), polysorbate 80 (Tween 80), olive oil, soybean oil, and 1-octanol were purchased from Sigma-Aldrich (St. Louis, MO, USA). Itraconazole, formic acid, LC-MS grade acetonitrile (ACN) and water were purchased from VWR Chemicals BDH^®^ (Radnor, PA, USA). Labrasol^®^, and Labrafac Lipophile WL1349^®^ were gifts from Gattefosse (Lyon, France). ACD/Labs (Toronto, Canada) was used to generate *in silico* physicochemical predictions for the compound.

Drug-free heparinized rat plasma was purchased from Biochemed Services (Winchester, VA, USA). Male Sprague Dawley rats (250–300 g) were purchased from Envigo (Indianapolis, IN, USA). Heparin (1000 units/mL) and pharmaceutical-grade normal saline were purchased from Hospira (Lake Forest, IL). VWR 200 Homogenizer was purchased from Avantor Inc. (Radnor, PA, USA).

### Methods

#### Analytical Assay

UPLC (Ultra Performance Liquid Chromatography) and LC-MS/MS assays were developed to quantify GT-14 from *in vitro* and *in vivo* studies, respectively. Samples from physicochemical and *in vitro* metabolism studies were analyzed by using the UPLC method. Samples from pharmacokinetics and tissue distribution studies were analyzed by a validated LC-MS/MS method (10).

#### UPLC Analysis of GT-14

A simple, sensitive, and reliable UPLC method with UV detection was developed for the quantification of GT-14. UPLC assay was developed using Waters ACQUITY UPLC H-class PLUS system. Baseline resolution was achieved using an AQUITY UPLC BEH C18 column (2.1 × 50 mm, 1.7 μm particle size) purchased from Waters Corporation (Milford, MA, USA), The optimized assay method used gradient elution with mobile phases consisting of water (0.1% formic acid) as mobile phase A and ACN as mobile phase B. The injection volume was 5 μL, and quantification was done at 305 nm. Itraconazole was used as internal standard (IS) at a concentration of 10 μg/mL. Stock solutions of GT-14 and itraconazole (IS) (Fig. 1b) were prepared by dissolving in ACN at a concentration of 1 mg/mL and kept at 4°C. Calibration curves for GT-14 were generated freshly on the days of sample quantification in the concentration range of 0.15–10 μ g/mL.

#### GT-14 Quantification by LC-MS/MS

The LC-MS/MS method used to quantify GT-14 has been described in a previous publication (10). Briefly, the assay was performed using a 6500+ Triple Quad LC-MS/MS System equipped with an Exion LC UHPLC unit (SCIEX LLC, CA, USA). GT-14 (*m/z* 265.2 → 134.1) and griseofulvin (Internal Standard, IS) (*m/z* 353.1 → 285.1) (Fig. 1c) were detected in a positive mode by electrospray ionization (ESI) using multiple reaction monitoring (MRM). Chromatographic separation was achieved using an AQUITY UPLC BEH C_18_ column (2.1 × 50 mm, 1.7 μm particle size) purchased from Waters Corporation (Milford, MA, USA). The optimized assay method used gradient elution with mobile phases consisting of water (0.1% formic acid) as mobile phase A and ACN (0.1% formic acid) as mobile phase B.

#### Preparation of Stock and Standard Solutions

The stock solutions of GT-14 and IS in ACN were prepared at 0.5 mg/mL and 1 mg/mL, respectively. The working standard solutions of GT-14 and Griseofulvin (IS) were prepared in ACN: water (50:50 v/v). Stock solutions of GT-14 and IS were stored at −20°C. Working standard solutions and quality control samples were freshly prepared.

#### Sample Preparation for Rat tissue

Each tissue sample was weighed and homogenized in a clean scintillation vial with 0.9% saline (1:1, w/v) using the VWR 200 Homogenizer to make tissue homogenates. Homogenization was carried out on ice at a speed of 5000 rpm for all tissues except the prostate and kidney (10,000 rpm). A five-point calibration curve was prepared for the different tissues in the range of 0.39–25 ng/g. One hundred (100) μL of tissue homogenate samples were mixed with 10 μL of Griseofulvin (1.5 μg/mL, IS). The samples were vortexed for 30 seconds which was followed by protein precipitation with methanol (300 μL), to extract GT-14. The samples were again vortexed for 30 seconds, and then shaken on an automatic shaker for 5 minutes, followed by centrifugation at 14000 rpm for 20 minutes at 4°C. The supernatant was transferred to a sample vial, and a 5 μL aliquot was injected for LC-MS/MS analysis.

#### Sample Preparation for Rat Plasma

An eight-point calibration curve was prepared in the range of 0.78 – 1,000 ng/mL. Twenty (20) μL of drug-free heparinized rat plasma was spiked with 2 μL working standard solutions of GT-14 and IS (final concentration of 50 ng/mL) to prepare standard solutions for constructing the calibration curve. Protein precipitation with methanol (200 μL) was used for the extraction of GT-14 from plasma. The extracted samples were vortexed for 30 seconds, shaken on an automatic shaker for 5 minutes, and centrifuged at 14000 rpm for 20 minutes at 4°C. The supernatant was transferred to a sample vial, and a 1 μL aliquot was injected for LC-MS/MS analysis.

### Pre-formulation Studies

#### Ionization Constant (pKa) Determination

The Ionization constant of GT-14 was determined using the Pion Sirius T3 apparatus (Sirius Analytical Instruments Ltd., East Sussex, UK). It has an Ag/AgCl double junction reference pH electrode, a Sirius ±D-PAS spectrometer and a turbidity sensing device. The titration experiments were conducted in 0.15 M KCl solution under an argon atmosphere at a temperature of 25 ± 1°C. All tests were performed using standardized 0.5 M KOH and 0.5 M HCl as titration reagents. pKa value was measured using UV-metric and Fast UV titration methods.

#### Lipophilicity

The lipophilicity of GT-14 as indicated by the logP value (octanol–water partition coefficient) was determined by the traditional shake-flask method. Briefly, equal volumes (1 mL) of 1-octanol and water were added to an amber glass bottle and allowed to equilibrate on a shaker for 24 h. Following equilibration, approx. 1 mg of GT-14 (dissolved in Acetonitrile) was added to the two-phase system and allowed to shake continuously at room temperature for 72 h. The two phases were separated using a separating funnel and then centrifuged at 15,000 rpm for 10 min. The concentration of GT-14 in the octanol and water phases was determined by UPLC assay. The experiment was conducted in triplicate.

The logP was calculated by the equation:

$$\text{log}P=\text{log}\frac{\text{OctanolConc}.}{\text{AqueousConc}.}$$

#### Solubility

Solubility of GT-14 was assessed in different solvents, namely, water, ethanol, PEG 300, PEG 400, propylene glycol, DMSO, DMA, olive oil, soybean oil, Labrasol and Labrafac, by the shake-flask method. Briefly, an excess amount of GT-14 was added to each of the selected solvents in a scintillation vial. The vials were allowed to shake on a mechanical reciprocating shaker at room temperature for 24 h to achieve solvent saturation. The samples were then centrifuged at 15,000 rpm for 15 min at room temperature. The supernatant with dissolved GT-14 was separated from the pellet of the undissolved compound. It was then diluted and analyzed by UPLC to determine the concentration of GT-14. All experiments were conducted in triplicates.

#### The pH-dependent Solubility and Stability

The pH-dependent stability of GT-14 was assessed in aqueous media of pH ranging from 1 to 10 at room temperature. Standard USP buffers (11) were prepared and used for this experiment. Details of buffer solutions are provided in Table I. For the pH solubility profile, GT-14 solid was added in excess amounts in various buffer solutions. Each buffer solution was prepared in triplicate. pH solubility profile of GT-14 was measured at 24 h.

To determine the stability of GT-14 in different buffers, GT-14 stock solution (prepared in ACN at a concentration of 75 μg/mL) was added to different buffers to achieve a target concentration of 5 μg/mL. Each buffer solution was prepared in triplicate. All solutions were put on the shaker bath maintained at 25°C. Aliquots were withdrawn at predetermined time points (0, 24, 72, 96, and 120 hrs), centrifuged at 15,000 rpm for 10 min, diluted with Water: ACN (50:50, v/v), and analyzed by the UPLC assay.

### Cosolvent Formulation and Stability

Co-solvent systems with various compositions and ratios of PEG 400, PEG 300, and propylene glycol were prepared to solubilize 5–10 mg/mL of GT-14. Each system was diluted with normal saline at different ratios of 1:1, 1:3, 1:7, and 1:15 (v/v) and observed for signs of precipitation for 4 h. The cosolvent system with maximum solubility and aqueous tolerance was tested for stability at different temperatures (−20°C, 4°C, and room temperature) in amber glass vials for up to a month. Samples were aliquoted at different time points and analyzed with UPLC.

### *In Vitro* Absorption and Metabolism Studies

#### Transport Study Using Caco2 Cell Culture

Caco-2 cells were cultivated as described previously with slight modification (12). Briefly, a cell monolayer was prepared by seeding 400,000 cells per insert (Nunc, surface area 4.2 cm^2^, 3 μm pore size). Cells were maintained at 37°C under 90% humidity and 5% CO_2_. Monolayers were used between 19 and 22 days after seeding. The integrity of each monolayer was checked by measuring the transepithelial electrical resistance (Millicell ERS^®^) before the experiment. The normal TEER values obtained were above 400 Ω·cm^2^. Cell monolayers with TEER values less than 400 Ω·cm^2^ were not used. Hank’s balanced salt solution (HBSS) (9.8 g/L) was supplemented with NaHCO_3_ (0.37 g/L), HEPES (5.96 g/L), and glucose (3.5 g/L) for all experiments, after adjusting the pH to the desired value.

For transport studies, 2.5 mL of GT-14 testing solution (10 μM) in HBSS (pH 7.4) was added to the apical side (A to B transport) or the basolateral side (B to A transport). Five donor samples (200 μL) and five receiver samples (200 μL) were taken at 0, 1, 2, 3, and 4 h followed by the addition of 200 μL of GT-14 test solution to the donor or 200 μL of blank HBSS buffer to the receiver side. Then, 100 μl of an ACN containing internal standard was added to the samples right after sampling. The TEER was measured at the end of the study, and the cell layer was washed carefully using cold blank HBSS 3 times, then cut and sonicated for 30 min in 1.0 mL of blank HBSS for intracellular GT-14 concentration measurement. All samples were centrifuged at 15,000 rpm for 15 min at 4°C for UPLC analysis.

The apparent permeability coefficient and efflux ratio were obtained according to the following equations:


$$P_{\text{app}}=\frac{\text{dQ}/\text{dt}}{{\text{AC}}_{0}}$$

***P***_***app***_, apparent permeability coefficient.

***dQ/dt*,** the permeability rate.

***A*,** the surface area of the cell monolayer.

***C***_***0***_, the initial concentration in the donor compartment.

***P***_***a-b***_, permeability coefficient from apical to basolateral.

***P***_***b-a***_, permeability coefficient from basolateral to apical.

#### Biotransformation Studies of GT-14: Phase I and Phase II

The *in vitro* phase I reaction was conducted using rat liver microsome, which contain the cytochrome P450 monooxygenases that catalyze oxidation, reduction, or hydrolysis reactions to introduce or expose functional groups on the substrate. NADPH is the cofactor to provide the reducing equivalents required for enzymatic activity, while the cofactor regeneration systems (NADP^+^, glucose-6-phosphate, and glucose-6-phosphate dehydrogenase) maintain a sustained supply of NADPH during the reaction to facilitate the metabolite, Potassium phosphate buffer (KPI) mimics physiological conditions. The components were added to microcentrifuge tubes in the following order: The stock solution GT-14 (5 μL, 1 mM) was pre-incubated with rat liver microsomes (12.5 μL, 20 mg/mL, Corning Inc.) at 37°C in KPI buffer(452.5 μL, 50 mM) for 5 min before the reaction was initiated by the addition of an NADPH regenerating system, which consisted of 25μL of solution A1(1.3 mM NADP+, 3.3 mM glucose-6-phosphate, 3.3 mM magnesium chloride), and 5μL of solution B1 (0.5 units/mL glucose-6-phosphate dehydrogenase). Reaction mixtures were incubated at 37 °C in a shaker bath, shaking at 100 rpm. Aliquots (100 μL) were withdrawn at 0, 1.5, 3, 5, and 24 h, and the reactions were terminated by adding 50 μL stop solution (ACN with 0.6% formic acid containing IS). The quenched samples were vortexed, centrifuged (14,000 rpm for 15 min) and analyzed by UPLC.

The *in vitro* phase II glucuronidation reaction was carried out using a published protocol that involves the use of uridine diphosphate glucuronic acid (UDPGA) as cofactor, saccharolactone as beta-glucuronidase inhibitor to drive the forward reaction to release the metabolite, potassium phosphate buffer (KPI) to mimic physiological conditions, and alamethicin as a surfactant for the response. The components were pipetted into microcentrifuge tubes in the following order: GT-14 testing solution in KPI (pH 7.4), solution B (25 mM saccharolactone and 5 mM magnesium chloride), solution A (25 mM uridine diphosphate glucuronic acid and potassium chloride), microsome. Samples (100 μL) were taken at 2 h. The reaction was terminated with 50 μL of solution containing 0.6% formic acid in acetonitrile + internal standard (rutin) at each time point and vortexed for 1 min. The samples were then centrifuged at 14,000 rpm for 15 min. The supernatant (100 μL) was then collected and loaded into the UPLC for analysis.

#### Pharmacokinetics and Tissue Distribution Studies

Healthy male Sprague Dawley rats (250–300 g) were used to evaluate the plasma pharmacokinetics and tissue distribution of GT-14, with approved animal protocol from the Institutional Animal Care and Use Committee (IACUC) at Texas Southern University. The jugular vein of rats was cannulated in-house the day before intravenous dosing and blood sample collection. GT-14 was administered intravenously as a co-solvent formulation via the jugular vein cannula at a dose of 5 mg/kg. Serial blood samples (100 μL) were collected up to 24 h post-dose. The blood samples were centrifuged at 3000 rpm for 10 min at 4°C, and plasma was collected and stored at −80°C till analysis. Urine samples were also collected at intervals of 0–7 and 7–24 hrs. using metabolic cages and stored at −20°C until analysis. At 24 hr, animals were sacrificed and organs such as prostate, liver, lungs, spleen, brain, kidneys, and heart were collected, following whole body perfusion (transcardiac perfusion) with normal saline to reduce residual blood in tissues. Plasma and tissue homogenate samples were processed (described in section “Analytical Assay”) and analyzed by LC-MS/MS. Tissue concentrations were calculated using the following formula:

$$\text{Tissue}\;\text{concentration}\;\left(\text{ng}/\text{g}\right)={\text{C}}_{\text{HTS}}\times {\text{V}}_{\text{HTS}}/{\text{W}}_{\text{TS}}$$

where, C_HTS_ is the concentration (ng/mL) of GT-14 in the homogenized tissue sample; V_HTS_ is the volume of homogenized tissue sample (mL) and W_TS_ is the weight of tissue sample (g).

## Results

### Analytical Assay

#### UPLC Assay

**Fig. 3:**
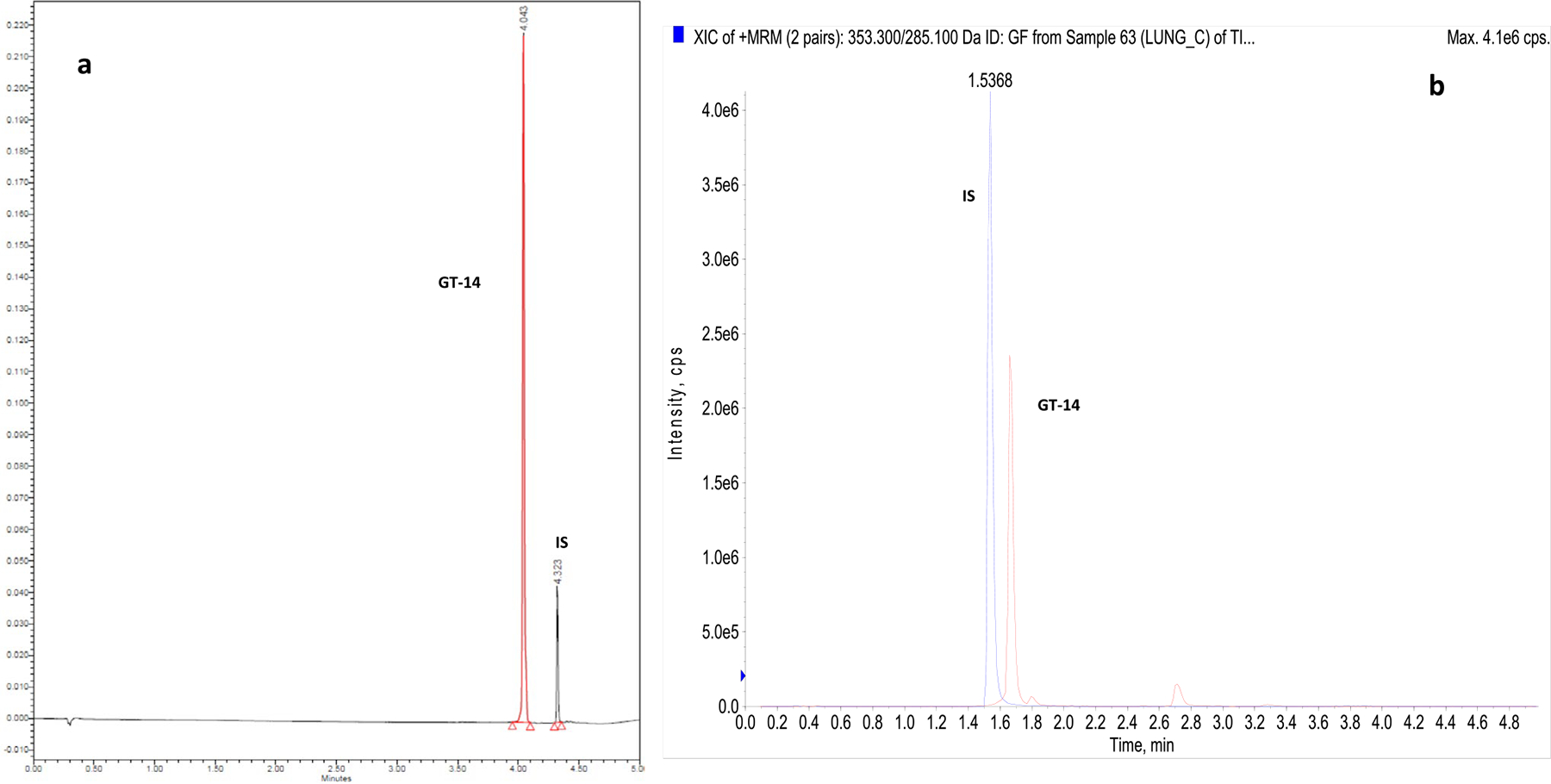
**a** Representative UPLC Chromatogram showing GT-14 (Rt=4.03 min) and IS (Rt=4.32 min); **b** Representative LC-MS/MS Chromatogram showing GT-14 (Rt=1.68 min) and IS (Rt=1.53 min) in tissue homogenate, respectively

A specific, accurate, and precise UPLC assay method with UV detection was developed and validated for the quantification of GT-14 in *in vitro* samples, where the drug concentrations are relatively high. GT-14 and IS were quantified at λ_max_ of 305 nm, respectively. Retention times of GT-14 and Itraconazole (IS) were 4.03 and 4.32 minutes, respectively. No interfering peaks were observed in the chromatograms of blank samples (Fig. 3a). Linearity was observed over the concentration range of 0.15 (LLOQ) −10 μg/mL with a coefficient of correlation greater than 0.999.

#### LC-MS/MS Assay

A sensitive and selective LC-MS/MS method was developed and validated, and previously reported for the quantitation of GT-14, in rat plasma (10). This method was utilized for the quantification of GT-14 in rat tissues (liver, lung, kidney, heart, brain, and prostate), after partial validation (Fig. 3b). The method was linear in the range of 0.39 to 25 ng/mL in the tissue homogenates.

### Pre-formulation Studies

#### Ionization Constant (pKa) Determination

The acid-base dissociation constant (pKa) of a compound is specific to its chemical structure. pKa is the pH of the medium where a monoprotic drug is 50% ionized and the tendency of a drug (molecule or ion) to keep a proton (H+) at its ionization center(s) (13). pKa is a key physicochemical parameter that has a significant impact on several biopharmaceutical characteristics, such as lipophilicity, solubility, protein binding, and permeability, which in turn directly affect pharmacokinetic (PK) characteristics such as absorption, distribution, metabolism, and excretion (ADME).

GT-14 exhibits dual pKas with values of 3.46 ± 0.014 and 10.53 ± 0.4. These values were comparable to the pKa values calculated by ACD/ChemSketch (4.5 ± 0.5 and 11.3 ± 0.3). The acidic pKa of 3.46 is contributed by the Nitrogen of the imine group, and the basic pKa of 10.53 is attributed to the phenolic group. With an isoelectric point of approximately 7.0, GT-14 is expected to act primarily as a weak acid at physiological or formulation pH (≈ 7–7.5). Consequently, formulation work—whether salt selection or dissolution modeling—should follow weak-acid principles, for example by converting the compound to a sodium or potassium salt to improve gastrointestinal solubility.


$$\text{pI}=\left[\text{pKa}\left(\text{acid}\right)+\text{pKa}\left(\text{base}\right)\right]/2$$

#### Lipophilicity

Lipophilicity is measure of a compound’s affinity for a lipophilic environment. It is a key physicochemical parameter that has significant impact on biological processes such as intestinal absorption, membrane permeability and partition to different tissues and organs, as well as the routes of drug clearance. It is most commonly represented as logP or logD, and is measured as the ratio at equilibrium of the concentration of a compound between two phases, an oil and aqueous phase. The logarithm of the n-octanol-water partition coefficient (logP) is frequently used as an indicator of lipophilicity in drug discovery(14).

The predicted logP value of GT-14, as calculated by ACD/Labs, is 3.40. We determined the logP value experimentally using the traditional shake flask method. The experimental logP value was calculated to be 2.38 ± 0.04, indicating GT-14 predominately distributed into the nonaqueous environment (Octanol phase) and, therefore, it can be expected to permeate the biological membranes with greater efficiency. The deviation between predicted and experimentally observed logP values can be attributed to several factors. The *in silico* ACD/logP model calculates logP based on the additive contributions of individual atoms, structural fragments, and intramolecular interactions among fragments. This constructionist approach begins by deriving basic fragment values from experimentally measured logP data of simple reference molecules. These values are then used to build a broader fragment set within the ACD/Labs internal database. When specific fragmental or intramolecular interaction contributions are unavailable in the database during logP prediction, secondary algorithms are employed to estimate them, introducing additional uncertainty into the prediction (15). Moreover, computational models do not typically account for solvent effects, pH variability, or other experimental conditions that can influence logP values. While *in silico* predictions are highly valuable in earlystage drug discovery, experimentally determined logP values remain more accurate and reliable.

#### Solubility

**Fig. 4:**
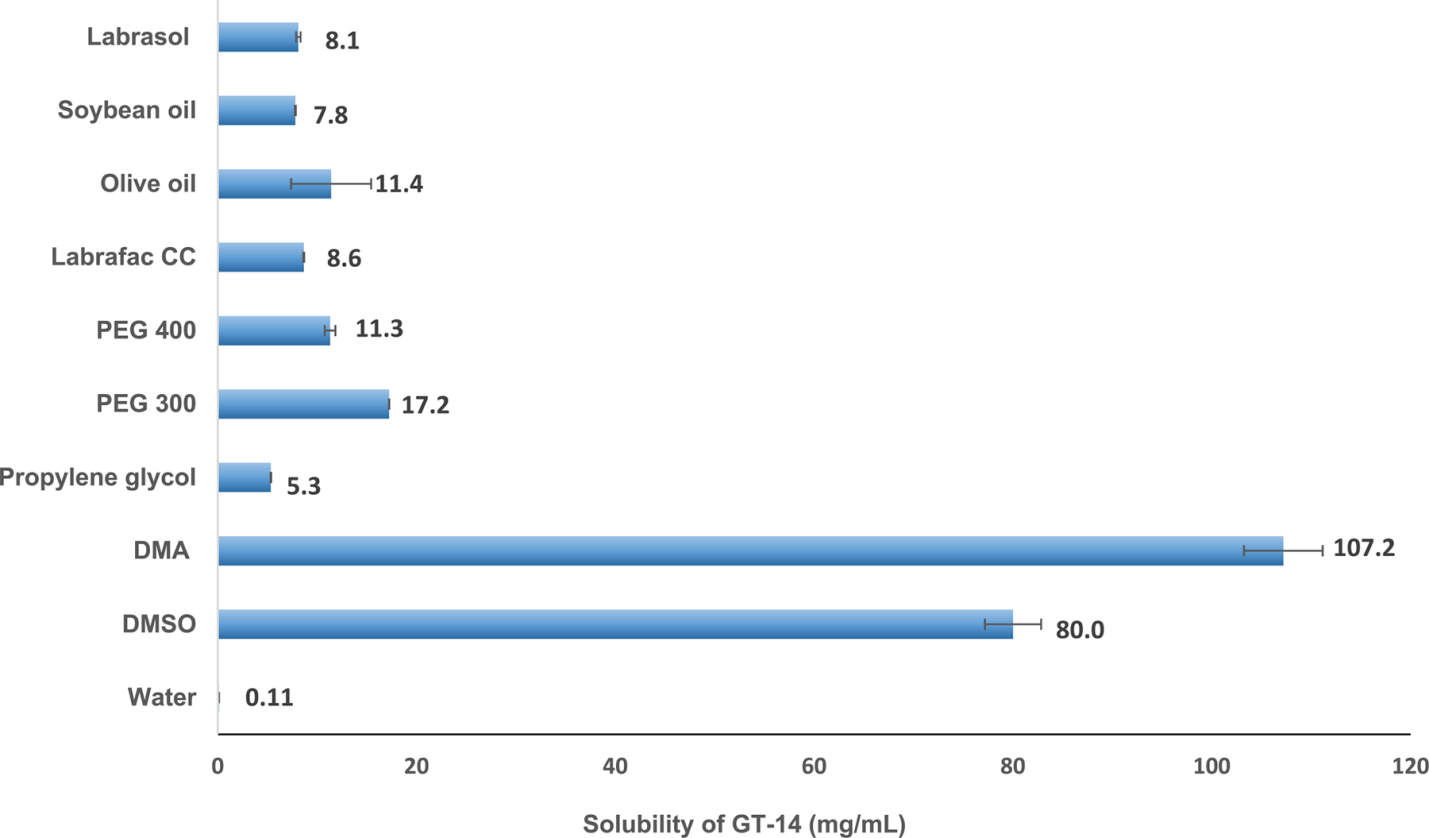
Solubility of GT-14 in different solvents (Mean± SD, *n* = 3 per solvent)

The water solubility of GT-14 was determined to be 0.11 mg/mL (Fig. 4), which indicates that GT-14 is very slightly soluble in water. Aqueous solubility is a crucial property that plays a role in almost every stage of drug development, influencing drug uptake, distribution, and elimination from the body. Compounds with low water solubility present significant challenges during drug discovery and development.

The effectiveness of a drug largely depends on its aqueous solubility; therefore, drugs with poor solubility or slow dissolution rates may be removed from the body before reaching the bloodstream, preventing them from exerting their intended pharmacological effects. Consequently, aqueous solubility is a key determinant of a drug’s oral absorption (16). Poor water solubility is a significant risk factor in low oral absorption because drug molecules must, in most cases, be in solution to be absorbed, and oral bioavailability is usually a required characteristic in a target product profile of an orally administered medicine (17). It is imperative to find the best possible solvents that can solubilize GT-14 and can be used for analytical method development and formulation development for PK studies. Several solvents (organic solvents, water miscible cosolvents, oils) were screened for solubility measurement of GT-14.

The solubility data are presented in Fig. 4, which shows that GT-14 is freely soluble in solvents such as DMSO and DMA., sparingly soluble in PEG 300, and PEG 400 and olive oil, and slightly soluble in propylene glycol, soybean oil, Labrafac Lipophile WL1349, and very slightly soluble in water.

#### pH Solubility and Stability Profile

**Fig. 5:**
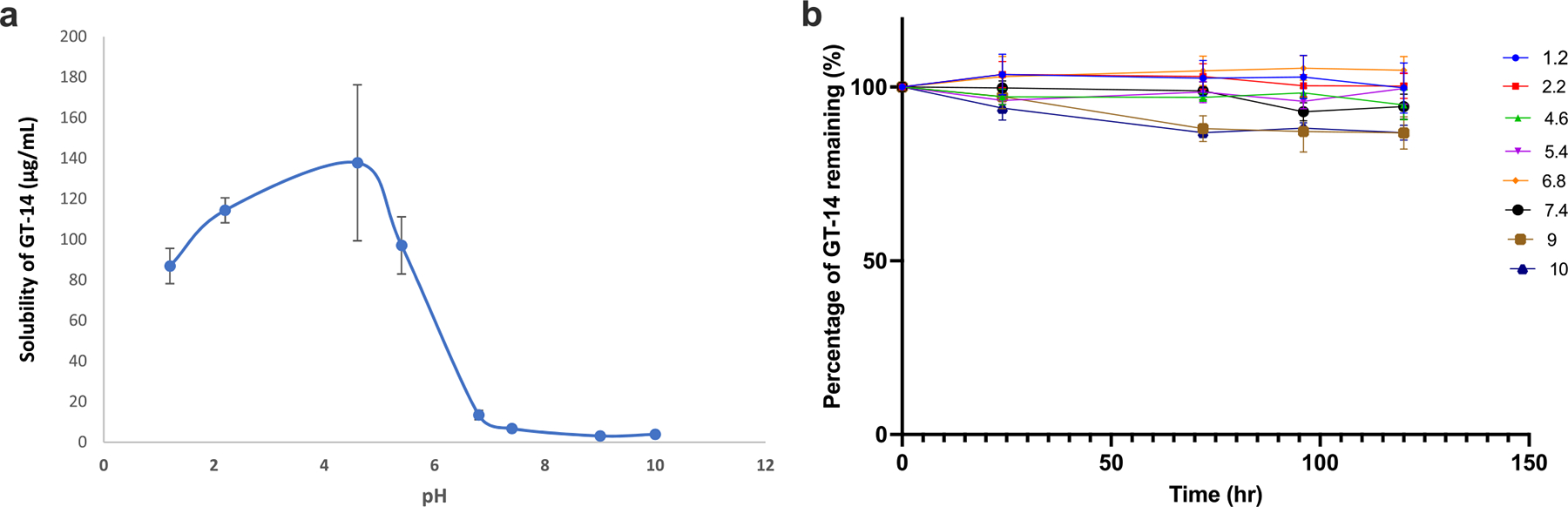
**a** pH solubility Profile of GT-14; **b** pH stability profile of GT-14 at 25°C (Mean ± SD, *n* = 3 per sample point)

An ionizable compound exhibits a distinct pH dependent solubility profile where the solubility at a given pH is determined by the free form of the drug and the counterion. Total solubility at a given pH is a function of both un-ionized and ionized species. At pH values greater than (for a weak acid) and less than (for a weak base) pKa, solubility increases due to the increased quantities of the ionized species in solution(17, 18). Solubility increases from ~80 μg/mL at pH 1.2 to ~140 μg/mL at pH 4.6 (Fig. 5a), consistent with protonation of the basic centre, which confers a net positive charge and enhances aqueous affinity. A steep drop in solubility occurs above pH 5, with levels falling below 10 μg/mL between pH 5 and 7. In this range, GT-14 exists mainly as a zwitterion; near its isoelectric point (pI ≈ 7.0), the net charge approaches zero, reducing electrostatic repulsion.

The stability of GT-14 was also studied over a period of 120 hours across a pH range of 1.2–10.0. at 25°C. As Fig. 5b shows, GT14 is relatively stable in acidic to neutral pH (1.2–7.4.) with minimal degradation over time. However, degradation is significantly pronounced in alkaline pH (pH 9–10), indicating that the compound is susceptible to base-catalyzed hydrolysis likely initiated by hydroxide attack on a labile functional group such as an ester or amide. These findings are critical in guiding formulation design, buffer selection, and storage conditions for maintaining compound stability. It is reasonable to believe that GT-14’s formulations should maintain a slightly acidic to neutral pH and avoid excipients that can elevate the pH, resulting in degradation.

#### Cosolvent Formulation Development and Stability

Based on the above preformulation studies, we successfully developed a cosolvent formulation of GT-14 with a combination of PEG 300, PEG 400, and propylene glycol for intravenous administration. Cosolvents are water-miscible organic solvents that enhance the solubility of poorly water-soluble drugs by lowering the polarity of the aqueous medium. This occurs by disrupting the hydrogen bonding network in water, creating a less polar environment that better matches the properties of nonpolar drug molecules. Common cosolvents such as propylene glycol, ethanol, PEG 300/400, and DMSO are widely used in parenteral drug formulations. Approximately 10–15% of FDA-approved parenteral products utilize cosolvents, often in high concentrations exceeding 50% (v/v). We developed a few cosolvent compositions to solubilize GT-14 (shown in Table II) and found our optimum formulation, which consisted of PEG 300, PEG 400, and propylene glycol in the ratio of 3:2:2 v/v. The solubility of GT14 in the formulation was 8 mg/mL, which is over 70 times the increase of GT-14’s original water solubility.

Cosolvents are commonly diluted before intravenous administration to reduce irritation at the injection site, but this can also increase the risk of drug precipitation. We evaluated the effect of dilution on precipitation by diluting the formulation with several ratios of saline. No precipitation was observed till a 10X dilution with saline. We also determined the physical and chemical stability of the optimum cosolvent formulation at different temperatures (−20°C, 4°C, and room temperature). Physical properties of the liquid formulation, such as appearance and color, were analyzed visually before the start of the chemical stability studies and at every sampling time point when the drug content was monitored. The initial appearance of the formulation was a light-yellow liquid without any visible particles. After one month, the formulation remained light yellow, without any visible particles, at −20°C and 4°C. At room temperature, the formulation grew a darker color. The GT-14 content in the formulation was measured using UPLC immediately after preparation (initial time) and at various time points over a month. The percentage of drug remaining at each time point is tabulated in Table III. Results indicate that formulation is stable at 4 °C and −20°C. At room temperature, the formulation is not very stable — the drug content declines to 93.4% on Day 7 and then further decreases to 90.8% in 30 days. The degradation and color change of the GT-14 cosolvent formulation at room temperature are likely due to oxidation caused by PEGs (19). PEGs and PG have high solubilizing power and are widely used to solubilize poorly soluble compounds. However, the hydroxyl group in PEGs is reactive and can result in the formation of degradation products. The peroxide impurities in PEG 400 are also known to promote significant degradation (20). Our study suggests that the solubility of GT-14 in the cosolvent formulation is > 98% at −20°C and 4°C, which is satisfactory and can be used for the pharmacokinetic studies.

### *In Vitro* Absorption and Metabolism Studies

#### Transport Study Using Caco2 Cell Culture

**Fig. 6:**
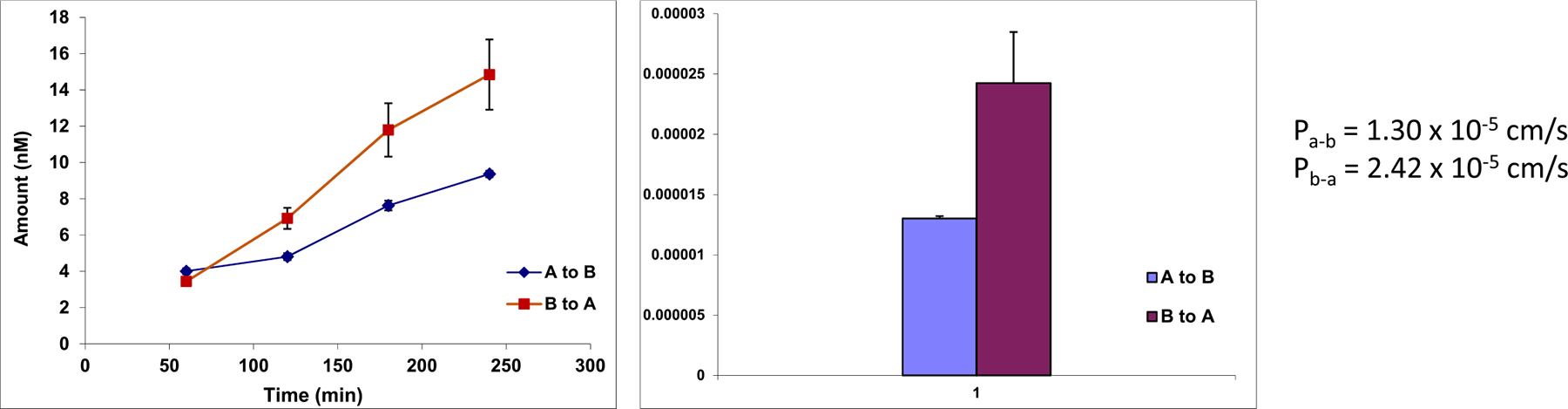
Bi-directional Transport of GT-14 in the Caco-2 cell culture model (each data point is represented as Mean ± SD, *n* = 3)

The bi-directional transport study of GT-14 (Fig. 6) showed that the apical to basolateral permeability (P_a-b_) and basolateral to apical permeability (P_b-a_) were 1.30 × 10^−5^ cm/s and 2.42 × 10^−5^ cm/s, respectively, when the donor side concentration was 10 μM. These high permeability values indicated that GT-14 has good permeation across the Caco-2 cell monolayers, predicting a good absorption (>75%) in the GI tract in humans(21). The P_b-a_ is higher (*p*<0.05, t-test) than P_a-b_. However, the efflux ratio is 1.8, indicating that efflux transporter(s), if present, are unlikely to affect *in vivo* absorption significantly according to FDA guidance.

#### Metabolic Biotransformation Reaction

**Fig. 7:**
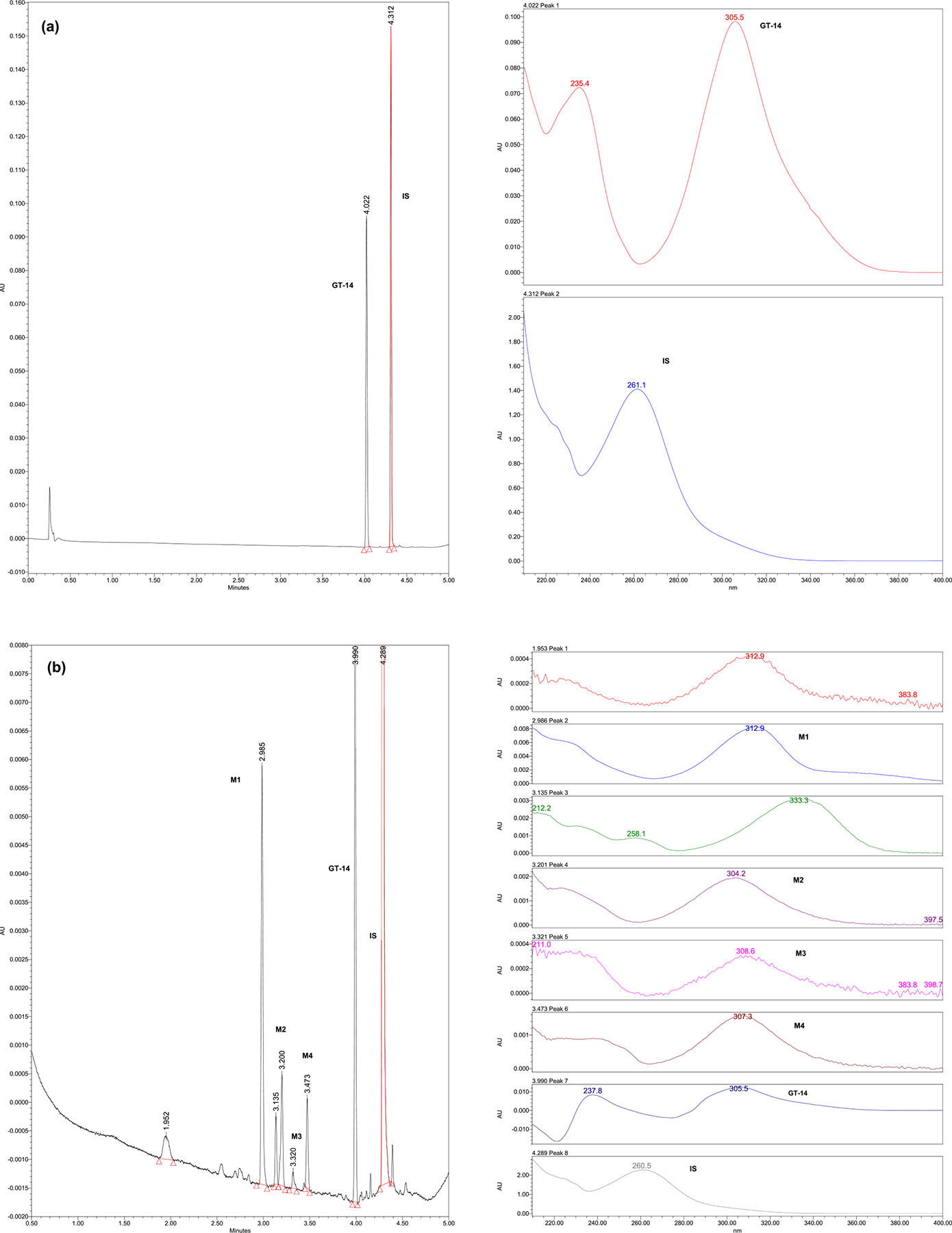
Chromatogram along with UV scans showing **a** GT-14 and IS at 0 hr **b** GT-14, internal Standard and phase I metabolites (M1, M2, M3 and M4)of GT-14 after 3 hr of incubation with rat liver microsomes

After incubation with liver microsomes in the phase 1 metabolism system, at least four additional peaks appeared. The UV spectra for additional peaks at 2.98, 3.20, 3.32, and 3.47 min were similar to that of GT-14 (Fig. 7), suggesting that GT-14 undergoes phase 1 metabolism. When GT-14 was incubated with liver microsomes in the glucuronidation reaction system, no additional peaks were observed, suggesting that glucuronidation might not be the major metabolism pathway for GT-14.

#### Pharmacokinetics and Tissue Distribution Study

The pharmacokinetics (PK) study was reported in a previous publication describing the LC-MS/MS method and its application (10). Briefly, following a single intravenous (IV) bolus administration of GT-14 at a dose of 5 mg/kg, the plasma concentration *vs*. time profile displayed a biphasic pattern. The drug was rapidly cleared from the systemic circulation within the first 60 minutes, followed by a slower, more gradual decline in plasma levels. PK parameters were derived using a two-compartment IV bolus model. The maximum plasma concentration (C_max_) of GT-14 was estimated to be approximately 200 ng/mL. The mean systemic clearance (CL) and (AUC_o–∞_) were calculated to be 229.9 mL/min/kg and 23,742 min*ng/mL, respectively. The distribution and elimination half-lives were 16.0 minutes and 268.1 minutes. The apparent volume of distribution (V_d_) was 25,205 ± 2,585 mL/kg, suggesting extensive tissue distribution beyond the central compartment (9).

GT-14 was not detected in the urine, indicating that renal excretion is not a primary route of elimination for the unchanged compound. GT-14 was found to be stable in rat urine (Supplemental Table 1). This suggests that GT-14 is primarily eliminated through metabolism and/or alternative excretory pathways.

**Fig. 8:**
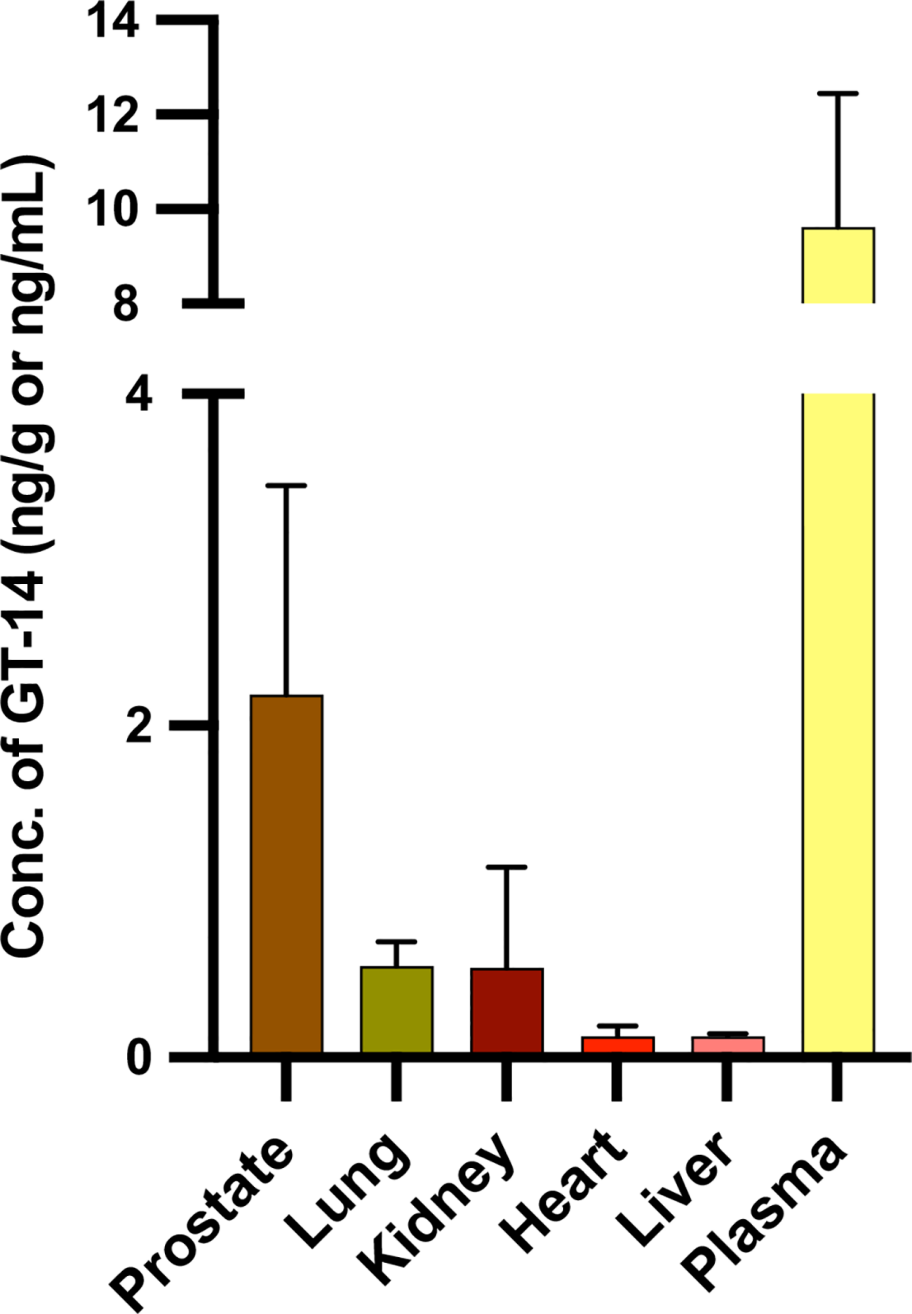
Tissue distribution profile of GT-14 (Mean ± SD, *n* = 3–4)

At 24 hr post-dose, GT-14 was detected in all sampled tissues except the brain. Tissue concentrations followed the order: prostate > kidneys > lungs > heart > liver. Notably, the prostate showed the highest accumulation, while low liver concentrations may be attributed to hepatic metabolism, as supported by prior *in vitro* metabolic studies (Fig. 8).

## Discussion

GT-14 is a first-generation small molecule inhibitor of Gα_i_2 protein, which is being developed for the treatment of mCRPC. Chemically it is a weakly basic phenolic imine with a molecular weight less than 500 (264.32 g/mol), LogP of less than 5 (logP=2.38), 1 hydrogen bond donor and 2 hydrogen bond acceptors. This set of molecular properties fulfills the requirements of Lipinski’s rule of 5, thus suggesting that GT-14 is orally deliverable. Lipinski’s ‘rule of five’ guides the design of ‘orally deliverable’ compounds and is based on the limits of molecular descriptors and physicochemical properties (clogP, molecular weight and number of hydrogen-bond donors and acceptors) beyond which oral activity is predicted to be poor.

The preclinical development of GT-14 followed an integrated blueprint (Fig. 2) that combined *in silico* predictions with experimental *in vitro* and *in vivo* studies. In this study, we performed integrated characterization of the physicochemical, metabolic, and pharmacokinetic properties of GT-14, providing valuable insights into its potential for further development. GT-14 exhibits an amphoteric solubility profile, with increased solubility in acidic conditions due to protonation of its basic group, peaking at pH 4.6. Solubility sharply declines between pH 5 and 7 as the molecule adopts a zwitterionic form. Its logP value of 2.38 ± 0.04 indicates favorable lipophilicity for membrane permeation, further supported by its high permeability across Caco-2 cell monolayers. Based on low aqueous solubility (0.11 mg/mL) and high permeability, GT-14 could be classified as a BCS class II molecule. The pH solubility profile is typical for an amphoteric compound. Further, stability studies indicate that GT-14 is stable at acidic to neutral pH (1.2–7.4) but undergoes significant degradation under alkaline conditions (pH 9–10), suggesting susceptibility to base-catalyzed hydrolysis, often observed for basic compounds. GT-14 undergoes Phase I metabolism as demonstrated from rat liver microsomal studies.

Pharmacokinetic analysis following IV bolus administration showed an initial rapid distribution phase followed by a slower terminal elimination phase from plasma. The two-compartment model provides a robust framework for describing this behavior. The initial rapid decline, known as the distribution phase (α-phase), is primarily attributed to the redistribution of drug from the central compartment (plasma and highly perfused organs) to peripheral compartment comprising of tissues with slower drug uptake (slow diffusion or slow perfusion of organs with high affinity for the drug) (22). As equilibrium is gradually established between plasma and tissue compartments, the rate of decline slows, transitioning into the elimination phase, where plasma concentration changes reflect systemic clearance. Drug distribution is a complex process influenced by physicochemical properties and physiological variables unique to each individual (22). Binding to proteins in tissues or plasma may influence the apparent volume of distribution and duration of action, but not the initial distribution kinetics (24).

GT-14 demonstrates a distinct tissue distribution profile characterized by relatively low overall tissue accumulation but preferential uptake in the prostate. Moreover, GT-14 was not detected in the brain and showed minimal hepatic accumulation, likely due to extensive hepatic metabolism. This could be due to the 24-h time point post single dose injection, where almost 99% of the drug has been cleared from plasma.

GT-14 showed significant accumulation in the prostate, highlighting its potential for targeted therapies in specific tissues. High inter-animal variability was observed in tissue concentrations, as shown in Supplemental Table 2. To normalize this, tissue-to-plasma ratios were calculated and found to be <1 across all tissues, indicating plasma levels exceeded those in tissues. To better understand GT-14’s preferential prostate distribution, we compared its PK and tissue distribution with drugs commonly prescribed for metastatic mCRPC, including antiandrogen deprivation therapy, chemotherapy, and targeted therapy. For instance, Enzalutamide, an androgen receptor inhibitor, exhibits dose-independent pharmacokinetics between 0.5–5 mg/kg (IV and oral). It is highly protein-bound (97–98%) and extensively distributed, with an apparent oral volume of distribution (Vd/F) around 110 L. Tissue-to-plasma ratios ranged from 0.406 (brain) to 10.2 (adipose) across 10 studied tissues. Enzalutamide is primarily metabolized by hepatic CYP2C8 and CYP3A4 and eliminated via feces (25, 26). Another non-steroidal antiandrogen, Bicalutamide, exhibits high oral bioavailability, long halflife (~25–30 hr), and undergoes hepatic metabolism with subsequent urinary and fecal excretion. [3H]-Bicalutamide distribution studies showed preferential accumulation in the prostate, pituitary gland, and seminal vesicles, with limited brain penetration (27). Abiraterone, a prodrug of abiraterone acetate, selectively and irreversibly inhibits CYP17. It is extensively protein-bound (95.6–99.9% to albumin; 89.4–95.6% to α1-acid glycoprotein) and widely distributed (Vd ~5630 L). While preclinical studies indicate it crosses the blood–brain barrier, human data is lacking (28).

Thus, comparative evaluation of GT-14 with currently approved therapies for mCRPC highlights significant differences in pharmacokinetic behavior and tissue distribution. Most approved agents exhibit rapid systemic clearance and extensive tissue penetration, including tumor and peripheral organs such as the liver and brain.

One of the primary challenges in achieving effective drug delivery to prostate tissue is overcoming the blood-prostate barrier (BPB). Structurally, the BPB is composed of tight junctions and specialized endothelial cells that tightly regulate the exchange of substances between the bloodstream and the prostate, thereby limiting the penetration of therapeutic agents. The translocation efficiency of compounds across the BPB is largely determined by their molecular weight and lipid solubility. GT-14, a low molecular weight and lipidsoluble compound, fulfills these criteria and is therefore likely to diffuse across the BPB and accumulate within the prostate. Notably, most prostatic diseases—including prostatitis, benign prostatic hyperplasia, and prostate cancer—disrupt the BPB, increasing its permeability and facilitating enhanced drug penetration. Several drugs, such as enzalutamide, an androgen receptor antagonist used in prostate cancer therapy, have demonstrated the ability to cross the BPB, as evidenced by favorable partition ratios (tissue-toplasma concentration). To further improve drug delivery across the BPB, various strategies have been explored, including intraprostatic injection, external physical forces (e.g., ultrasonic sonoporation and photodynamic therapy), biological vectors such as cell motors and cytokines, and nanotechnology-based delivery systems (29).

GT-14 is a selective inhibitor of the Gαi2 protein, which is broadly expressed across various human tissues, including the brain, endocrine glands, liver, reproductive organs, and immune cells, as reported by the Human Protein Atlas (30). Gα_i_2 plays critical roles in angiogenesis, cardiovascular regulation, dopaminergic signaling, and immune function. Given its widespread physiological importance, systemic inhibition of Gα_i_2 may lead to off-target toxicity. However, our tissue distribution study revealed that GT-14 was not detected in the brain, and its accumulation in other tissues was minimal, suggesting a favorable safety profile. However, more studies are warranted to further elucidate the safety profile of GT-14.

The preclinical roadmap (Fig. 2) guided us through various studies, informing decisions on GT-14 development. It operates as a data→model→decision framework, converting each experimental readout into advance/hold criteria. Early physicochemical definition (dual pKa, logP, pH-solubility/stability), with formulation screening, establishes whether expected exposure is technically achievable and stable under preclinical and clinical conditions. For GT-14, amphoteric ionization with low aqueous solubility supported a pragmatic cosolvent approach that maintained robustness in concentration and dilution. Validated UPLC and LC–MS/MS methods provide the bioanalytical foundation, yielding reliable PK/ADME concentrations so downstream models are built on vetted measurements rather than assay assumptions. *In-vitro* permeability and microsomal biotransformation for translational characterization supported the working clearance hypothesis - permeability is adequate and clearance is mainly metabolism-driven and - provided the rationale for *in-vivo* PK with tissue distribution to fix estimates of CL and Vd, mapping tissue preference. For GT-14, rapid distribution, large apparent Vd, nonrenal elimination, and prostate-preferential exposure suggest a focus on hepatic clearance risk while flagging potential pharmacological advantages worthy of subsequent investigation. The next step involves model-informed advancement of the drug candidate from preclinical to clinical stage with a credible human CL/Vd projection, and the safety margin is sufficient to justify a first-in-human dose. The preclinical data is crucial for this purpose. The current scope of our present study does not include the model-informed drug development stage.

To summarize our findings and to facilitate the go/no-go decision process, we have tabulated the observations based on the criteria of physicochemical properties, biopharmaceutics, *in vitro* ADME, and *in vivo* studies (Table IV). The number of “go” decisions is more than “no-go”. Additionally, solutions (e.g., co-solvent for enhanced aqueous solubility) for “no-go” are available. Therefore, we decided to continue developing GT-14. We acknowledge that further studies on physicochemical degradation and metabolic pathways are warranted to optimize dosing strategies and ensure the compound’s long-term stability and efficacy. GT-14 shows strong potential as a candidate for further formulation and therapeutic development, particularly with modifications to stabilize its formulation and enhance its pharmacokinetic and biodistribution properties. Our lab is currently working on the development of more advanced targeted delivery systems for GT-14.

## Conclusion

Preclinical drug development usually takes 3–5 years from the identification of a new chemical entity (NCE) to advance to clinical testing. Most PK/PD and formulation evaluations are required for IND applications. To achieve higher success rates and reduce wasted funds and effort, de-risking studies, including *in vitro* characterizations (physicochemical properties, metabolism, stability, and cellular and gastrointestinal permeability including transporters, stability, and formulation development) and *in vivo* evaluation (PK/PD studies, physiologically-based PK modeling, toxicokinetics) should be conducted at earlier stages in the drug discovery and development process. These studies are critical for exemplifying a ‘go’ or ‘no-go’ decision-making process in drug development, highlighting the importance of earlystage evaluations in effectively guiding a drug candidate’s progression from IND filing to clinical trials.

The preclinical development of GT-14 adhered to the blueprint outlined in Fig. 2, advancing this novel compound toward its potential as a preventive/therapeutic option for patients with advanced mCRPC.

## Supplementary Material

sup

**Supplementary Information** The online version contains supplementary material available at https://doi.org/10.1208/s12248-025-01166-y.

## Figures and Tables

**Table I:** USP Buffers Used in the pH-dependent Solubility and Stability Study of GT-14

pH	Buffer Type	Composition
1.2	Hydrochloric Acid Buffer	50 mL of 0.2 M KCl + 85.0 mL of 0.2 M HCl, dilute to 200 mL with water
2.2	Acid Phthalate Buffer	50 mL of 0.2 M potassium biphthalate + 7.8 mL of 0.2 M HCl, dilute to 200 mL
3.4	Acid Phthalate Buffer	50 mL of 0.2 M potassium biphthalate + 10.4 mL of 0.2 M HCl, dilute to 200 mL
4.5	Neutralized Phthalate Buffer	50 mL of potassium bipthalate + 11.1 mL of 0.2 M NaOH, dilute to 200 mL
6.8	Phosphate Buffer	50 mL of 0.2 M KH_2_PO_4_ + 22.4 mL of 0.2 M NaOH, dilute to 200 mL
7.4	Phosphate Buffer	50 mL of 0.2 M KH_2_PO4 + 39.1 mL of 0.2 M NaOH, dilute to 200 mL
9.0	Alkaline Borate Buffer	50 mL of 0.2 M boric acid + KCl + 20.8 mL of 0.2 M NaOH, dilute to 200 mL
10.0	Alkaline Borate Buffer	50 mL of 0.2 M boric acid + KCl + 43.7 mL of 0.2 M NaOH, dilute to 200 mL

**Table II:** Cosolvent Formulations of GT-14

Composition	Ratio	Solubility (mg/mL)
PEG 400: Propylene glycol: Tween 80	2:1:0.25	5
PEG300: PEG400: Propylene glycol	1:1:1	6
PEG300: PEG400: Propylene glycol	3:2:2	8

**Table III:** Stability of Cosolvent Formulation of GT-14

Storage condition	Percentage Recovery		
	Day 7	Day 15	Day 30
−20°C	100.32 ± 0.08	101.22 ± 0.27	101.26 ± 0.39
4°C	101.09 ± 2.19	99.14 ± 0.32	98.83 ± 0.15
Room Temperature	93.37± 0.76	91.18 ± 0.38	90.88 ± 0.07

**Table IV:** Go/No-go Decisions for Future Development of GT-14

Category	Decision Criteria	Actual Outcome	Status	Comments/Rationale
Physico-chemical Characteristics	API Solubility in aqueous media	0.11 mg/mL	□ No-Go	Very slightly soluble in water. Requires solubility enhancement strategy
	logP(Lipophilicity)	2.38	✓ Go	Moderate Lipophilicity
	pH stability in physiological range (pH 1.2–7.4) (upto 120 hours)	> 90% remaining	✓ Go	Stable across physiological pH range
Biopharmaceutics	Caco-2 permeability	1.3 × 10^−5^ cm/s	✓ Go	High permeability
Metabolism	Phase I and Phase II metabolism	Phase I metabolism	□ No-Go	Susceptible to metabolism; potential for fast clearance
Formulation for PK studies	Solubility of GT-14 in Co-solvent formulation	8 mg/mL	✓ Go	Solubility enhanced many folds
	Solubility of GT-14 in Co-solvent at different temperatures (1 month)	>95% remaining at 4°C and −20 °C	✓ Go	Satisfactory for PK dosing
*In Vivo* PK	Plasma half-life in rats	4–5 hrs	✓ Go	Satisfactory
	Tissue distribution	High accumulation in prostate	✓ Go	Reaches target site of action

## Data Availability

Data supporting the findings of this study are available upon request from the corresponding author.
