# Supplementary material for: Preclinical Development of GT-14, a Novel Inhibitor of Gαi2 Protein: Comprehensive Evaluation of Physicochemical, Metabolic Characteristics and Tissue Distribution
Source: AAPS J. Author manuscript; Available in PMC 2026 Apr 5. (PMC13050480; doi:10.1208/s12248-025-01166-y)
Supplement: sup [file NIHMS2157385-supplement-sup.docx]

**Supplemental Table 1: Stability of GT -14 in rat urine**

| **Conc. (ng/ml)** | **Stability at -20 ^o^C (48 hrs)** |
| --- | --- |
| 25 | 109.28 ± 2.57 |
| 50 | 110.25 ± 2.36 |
| 500 | 105.71 ± 3.76 |
| 1000 | 101.43 ± 2.58 |

Blank rat urine was spiked with GT-14 at conc. of 25, 50, 500 and 1000 ng/mL. The samples were kept at -20°C for 48 hours (n=5, Mean ± SD)

**Supplemental Table 2: Tissue concentrations of GT-14 in different tissues at 24 hr post dose**

| **Tissue** | **Conc. (ng/mL for plasma or ng/g for the tissues)** |
| --- | --- |
| Prostate | 2.19 $\pm1.26$ (57.5) |
| Lung | 0.55 $\pm0.15$ (27.3) |
| Kidney | 0.52 $\pm0.61 ($112.6) |
| Heart | 0.13 $\pm$ 0.06 (46.2) |
| Liver | 0.13 $\pm0.01$ (11.3) |
| Plasma | 9.62 $\pm$ 2.82 (29.3) |

Data is shown as Mean ± $SD (\%CV)$
